# Supplementary material for: Feeding Preferences of Giant Pandas May Reflect the Detection of Specific Volatiles and Bitter-Tasting Metabolites in Bamboo Leaves as Markers of Nutritional Status
Source: Plants (Basel). 2025 Dec 18;14(24):3856. doi: 10.3390/plants14243856 (PMC12737210; doi:10.3390/plants14243856)
Supplement: Supplementary file 1 [file plants-14-03856-s001.zip › plants-3949443-supplementary.pdf]

Supplement

1. Bamboo feeding

Table S1 the observation record of giant panda A eating bamboo

Name of bamboo	**Date 8.28**			**Date 9.4**			**Date 9.9**			**Date 9.11**		
	Feed volume （kg）	Food intake （kg）	Feeding sequence	Feed volume （kg）	Food intake （kg）	Feeding sequence	Feed volume （kg）	Food intake （kg）	Feeding sequence	Feed volume （kg）	Food intake （kg）	Feeding sequence
Bashania fargesii	2120	400	2	1360	280	2	1600	480	1	1880	620	2
Indocalamus tessellatus	2120	40	3	840	40	3	860	140	3	1300	160	3
Pseudosasa japonica	3200	480	1	2600	920	1	1560	480	2	1360	600	1
Phyllostachys bissetii	1960	0		960	40	7	1504	40	6	830	10	7
Phyllostachys aureosulcata	2280	80	4	1320	60	6	656	20	7	400	20	6
Phyllostachys vivax	1600	20	6	1560	60	5	2080	70	4	2440	120	5
Phyllostachy	2040	20	5	2200	200	4	1132	30	5	640	340	4

s propinqua												
Phyllostachys parvifolia	3200	0		3600	20		2800	0		2620	0	
Phyllostachys iridescen	1440	0		1580	0		3040	0		2940	0	
Phyllostachys praecox	2720	0		3600	0		2720	0		2560	0	

Table S2 the observation record of giant panda Meng B eating bamboo

Name of bamboo	**Date 8.28**			**Date 9.4**			**Date 9.9**			**Date 9.11**		
	Feed volume **（kg）**	Food intake **（kg）**	Feeding sequence	Feed volume **（kg）**	Food intake **（kg）**	Feeding sequence	Feed volume **（kg）**	Food intake **（kg）**	Feeding sequence	Feed volume **（kg）**	Food intake **（kg）**	Feeding sequence
Bashania fargesii	1840	660	1	1440	560	1	1960	720	1	2160	760	1
Indocalamus tessellatus	1210	720	3	960	920	3	2520	520	3	1960	440	3
Pseudosasa japonica	2830	1790	2	3280	2000	2	2480	2040	2	2800	1840	2
Phyllostachy	960	120	7	960	40	6	1320	440	5	1070	30	7

s bissetii												
Phyllostachys aureosulcata	880	145	6	880	50	7	400	225	7	640	120	5
Phyllostachys vivax	2400	320	4	2400	120	5	2910	450	4	2195	795	4
Phyllostachys propinqua	2720	240	5	2720	120	4	1815	255	6	630	40	6
Phyllostachys parvifolia	2630	0		1920	0		2120	0		2160	0	
Phyllostachys iridescen	2250	0		2080	0		2300	0		2380	0	
Phyllostachys praecox	2370	0		2880	0		2920	0		3040	0	

Table S3 the observation record of giant panda C eating bamboo

Name of bamboo	**Date 8.28**			**Date 9.4**			**Date 9.9**			**Date 9.11**		
	Feed volume （kg）	Food intake （kg）	Feeding sequence	Feed volume （kg）	Food intake （kg）	Feeding sequence	Feed volume （kg）	Food intake （kg）	Feeding sequence	Feed volume （kg）	Food intake （kg）	Feeding sequence
Bashania fargesii	2680	960	1	1240	320	1	2280	640	1	1500	800	1

Indocalamus tessellatus	1640	520	3	960	600	3	3348	348	3	1020	420	3
Pseudosasa japonica	3400	400	2	2080	1680	2	1680	1280	2	2880	1140	2
Phyllostachys bissetii	2960	80	5	920	120		1480	20	7	1024	364	4
Phyllostachys aureosulcata	2400	0		1040	0		628	148	5	720	456	5
Phyllostachys vivax	1800	160	4	1520	200	5	2640	240	4	1308	12	7
Phyllostachys propinqua	2260	60	6	2160	160	4	1600	40	6	928	56	6
Phyllostachys parvifolia	3200	0		2040	20	6	2920	0		2380	0	
Phyllostachys iridescen	2000	0		1100	0		2840	0		2220	0	
Phyllostachys praecox	2920	0		2040	0		2740	0		2440	0	

Table S4 the observation record of giant panda D eating bamboo

Name of bamboo	**Date 8.28**			**Date 9.4**			**Date 9.9**			**Date 9.11**		
	Feed volume （kg）	Food intake （kg）	Feeding sequence	Feed volume （kg）	Food intake （kg）	Feeding sequence	Feed volume （kg）	Food intake （kg）	Feeding sequence	Feed volume （kg）	Food intake （kg）	Feeding sequence
Bashania fargesii	3000	1480	1	1370	825	1	2240	1480	1	1740	1480	1
Indocalamus	1640	1240	3	1430	1050	2	2740	1240	2	1125	1240	2

tessellatus												
Pseudosasa japonica	3200	200	2	2915	775	3	1850	200	3	2680	200	3
Phyllostachys bissetii	2060	140	7	1840	80	7	1360	140	7	1570	140	7
Phyllostachys aureosulcata	2650	140	5	2150	110	5	760	140	5	1175	140	5
Phyllostachys vivax	2200	100	6	1780	60	6	2410	100	6	1460	100	6
Phyllostachys propinqua	1840	120	4	1525	279	4	1640	120	4	1055	120	4
Phyllostachys parvifolia	2160	0		3260	0		2755	0		2745	0	
Phyllostachys iridescen	2175	0		2390	0		2640	0		2715	0	
Phyllostachys praecox	2900	0		2120	0		2535	0		2665	0	

Table S5 the observation record of four kinds of giant panda total amount of eating bamboo

**Name of bamboo**	**giant panda A**		**giant panda B**		**giant panda C**		**giant panda A**	
	**Feed volume （kg）**	**Food intake （kg）**	**Feed volume （kg）**	**Food intake （kg）**	**Feed volume （kg）**	**Food intake （kg）**	**Feed volume （kg）**	**Food intake （kg）**
Bashania fargesii	1740	445	1850	675	1925	680	2088	1316
Indocalamus	1280	95	1663	650	1742	472	1734	1193

Name of bamboo	giant panda A		giant panda B		giant panda C		giant panda A	
	Feed volume （kg）	Food intake （kg）	Feed volume （kg）	Food intake （kg）	Feed volume （kg）	Food intake （kg）	Feed volume （kg）	Food intake （kg）
tessellatus								
Pseudosasa japonica	2180	620	2848	1918	2510	1125	2661	344
Phyllostachys bissetii	1314	23	1078	158	1596	146	1708	125
Phyllostachys aureosulcata	1164	45	700	135	1197	151	1684	133
Phyllostachys vivax	1920	68	2476	421	1817	153	1963	90
Phyllostachys propinqua	1503	148	1971	164	1737	79	1515	160
Phyllostachys parvifolia	3055	5	2208	0	2635	5	2730	0
Phyllostachys iridescen	2250	0	2253	0	2040	0	2480	0
Phyllostachys praecox	2900	0	2803	0	2535	0	2555	0

Table S6 volatile components of 10 bamboo, the staple food of giant panda

序号	Name of component	*Bashania fargesii*	*Pseudosasa japonica*	*Indocalamus tessellatus*	*Phyllostachys bissetii*	*Phyllostachys aureosulcata*	*Phyllostachys vivax*	*Phyllostachys propinqua*	*Phyllostachys parvifolia*	*Phyllostachys iridescen*	*Phyllostachys praecox*
1	1-Penten-3-ol	1.30	1.64	0.91	2.97	1.76	1.41	1.74	0.30	1.41	0.20
2	2-Penten-1-ol, (Z)-	0.65	0.14	-	0.61	0.63	0.31	-	-	0.42	0.31
3	3-Buten-2-ol, 2-methyl-	-	-	-	0.81	-	0.90	-	0.06	1.68	0.46
4	3-Pentanone	4.87	9.90	8.94	-	-	10.80	-	5.10	9.26	4.16
5	Acetone	9.12	8.60	2.66	17.22	10.95	14.93	7.18	5.36	9.32	8.10

6	Benzene, 1-ethenyl-4-methoxy-	0.77	2.40	-	6.66	0.76	3.51	0.95	0.21	1.59	0.28
7	Cyclotetrasiloxane, octamethyl-	-	-	-	0.34	-	-	-	-	-	0.13
8	Cyclotrisiloxane, hexamethyl-	0.43	0.23	1.10	1.03	0.70	0.92	0.49	0.84	0.32	0.56
9	Disiloxane, hexamethyl-	7.85	6.62	17.04	12.39	18.49	14.37	13.34	13.63	7.67	10.20
10	Furan, 2-ethyl-	15.98	16.33	10.26	7.56	11.06	7.98	3.56	4.50	10.19	6.99
11	Furan, 2-methyl-	15.46	6.28	6.01	2.91	9.90	5.26	2.16	4.97	6.59	14.17
12	Heptane, 2,2,4,6,6-pentamethyl-	5.78	2.72	4.26	4.73	5.41	5.66	7.33	8.74	8.52	3.11
13	Silanol, trimethyl-	3.91	3.82	4.13	6.04	6.43	6.51	4.89	3.44	2.79	1.75
14	Trisiloxane, octamethyl-	0.23	0.11	1.06	1.07	0.72	0.66	0.53	0.76	0.29	0.59
15	1-Hexanol	0.89	1.04	1.03	2.14	2.10	2.00	1.59	-	0.12	1.08
16	2-Penten-1-ol, (E)-	0.51	-	0.18	-	-	0.43	-	0.57	0.37	0.48
17	3-Ethyl-2-nonone	0.40	-	0.22	0.50	-	-	0.43	-	0.37	0.19
18	Bicyclo[2.2.1]heptan-2-one, 1,7,7-trimethyl-, (1S)-	-	-	0.54	3.05	-	-	1.39	0.14	-	0.13
19	2-Pentanol	-	0.93	1.19	-	-	-	0.52	0.45	0.26	-
20	1,4-Dioxin, 2,3-dihydro-	2.14	3.25	5.89	0.44	-	0.77	4.42	2.44	0.84	0.40
21	2-Pentene, (E)-	0.52	0.35	0.37	0.14	0.23	0.57	0.54	0.30	-	-
22	Ethanol	1.18	10.15	9.61	8.96	4.05	2.14	7.43	23.45	10.99	35.35
23	1-Butanol, 3-methyl-	-	0.85	2.64	0.29	1.16	0.60	2.95	1.71	1.81	0.52
24	1-Pentene	-	0.33	0.16	0.46	0.34	-	-	0.51	0.30	0.29
25	1,3-Pentadiene	0.85	0.51	0.29	0.87	0.53	0.70	0.39	1.02	0.84	0.45
26	2-Butene, 2-methyl-	0.63	0.79	0.31	1.23	0.50	1.13	0.63	1.25	0.77	0.84
27	2-Pentene, (Z)-	0.69	2.02	0.72	2.25	1.69	0.53	2.47	1.24	1.37	1.68
28	2,2,4,4-Tetramethyloctane	0.72	0.15	0.36	0.45	0.61	0.67	1.13	1.10	1.00	0.39
29	Furan, 2-pentyl-	-	-	-	0.34	-	-	0.09	0.53	0.26	-
30	1-Propene, 2-methyl-	-	-	0.16	0.35	-	-	-	-	-	0.16
31	1,3-Pentadiene, (Z)-	-	-	-	0.70	0.18	0.45	0.10	-	0.12	-
32	2-Hexanone, 3,4-dimethyl-	-	-	-	0.25	-	-	-	0.45	0.34	0.26

33	Benzene, 1-ethyl-4-methoxy-	-	0.51	2.18	0.51	-	0.29	-	-	0.50	-
34	Pentane, 3-methyl-	0.32	0.14	-	0.15	0.33	-	-	0.19	-	-
35	1,3-Pentadiene, (E)-	0.33	0.12	-	-	-	-	-	0.13	0.18	-
36	Furan, 3-methyl-	0.09	1.21	-	-	-	-	7.87	-	-	0.08
37	s-Hydroxymethylthiobenzoate	0.25	-	-	-	-	-	0.59	-	-	0.16
38	1-Butanol, 2-methyl-, (S)-	-	-	0.57	-	-	0.27	0.27	0.13	0.43	-
39	1-Butanol, 2-methyl-	-	0.24	0.87	-	-	-	0.56	-	-	0.08
40	Oxirane, (1-methylbutyl)-	-	2.04	-	-	-	-	-	1.21	-	-
41	Silane, methyl-	-	0.28	0.53	-	-	-	-	-	0.52	0.08
42	2-Hexel	9.99	-	-	1.62	-	1.63	-	-	-	-
43	Benzaldehyde	0.14	-	0.25	0.25	2.43	1.25	0.54	-	-	-
44	Phenol, 2-ethyl-5-methyl-	-	0.24	-	0.53	-	-	0.43	-	-	-
45	(1R)-2,6,6-Trimethylbicyclo[3.1.1]hept-2-ene	1.20	-	-	1.56	-	-	-	-	-	-
46	Cyanic acid, sec-butyl ester	0.19	-	-	0.17	-	-	-	-	-	-
47	(R)-(-)-2-Pentanol	0.14	0.11	-	-	-	0.53	-	-	-	-
48	1,3-Cyclobutanediol, 2,2,4,4-tetramethyl-	0.15	-	-	-	0.11	-	-	-	-	-
49	1,2:5,6-Dianhydrogalactitol	-	-	0.83	-	-	-	0.35	-	-	-
50	Oxirane, (methoxymethyl)-	-	-	0.27	-	-	-	0.66	-	-	-
51	5-Octyn-3-ol	-	-	0.31	-	-	-	0.33	-	-	-
52	1-Benzoyl-3-amino-4-cyano-3-pyrroline	0.20	-	-	-	-	-	-	-	-	-
53	1,3-Propanediol	0.82	-	-	-	-	-	-	-	-	-
54	N-Dimethylaminomethyl-tert.-butyl-isopropylphosphine	2.17	-	-	-	-	-	-	-	-	-
55	2-Methyl-1-butene	-	-	0.71	-	-	-	-	-	-	-
56	Carbonyl sulfide	-	-	0.25	-	-	-	-	-	-	-

57	Methanediamine, N,N,N',N'-tetramethyl-	-	-	0.47	-	-	-	-	-	-	-
58	.alpha.-Pinene	-	2.45	2.59	-	-	-	-	-	-	-
59	2-Butanol, 3-methyl-	-	-	4.86	-	-	-	-	-	-	-
60	Bicyclo[3.1.1]heptane, 6,6-dimethyl-2-methylene-, (1S)-	-	-	0.55	-	-	-	-	-	-	-
61	Camphene	-	-	0.60	-	-	-	-	-	-	-
62	Tricyclo[2.2.1.0(2,6)]heptane, 1,7,7-trimethyl-	-	-	0.20	-	-	-	-	-	-	-
63	2,5-Dihydro-3-methyl-thiophene 1,1-dioxide	-	1.01	-	-	-	-	-	-	-	-
64	Dimethyl ether	-	9.25	-	-	-	-	-	-	-	-
65	Butanenitrile, 2-methyl-	-	-	-	0.35	-	-	-	-	-	-
66	Hydrazine, 2-butenyl-	-	-	-	0.53	0.17	0.52	-	-	-	-
67	Methacrolein	-	-	-	-	0.68	-	-	-	-	-
68	Valine	-	-	-	-	0.73	-	-	-	-	-
69	Cyclobutanol, 2-ethyl-	-	-	-	-	-	0.18	-	-	-	-
70	1,3-Pentadiene, 2,4-dimethyl-	-	-	-	-	-	-	11.44	-	-	-
71	Cyclopropane, propyl-	-	-	-	-	-	-	0.08	-	-	-
72	Decane, 2,2-dimethyl-	-	-	-	-	-	-	1.64	-	-	-
73	Cyclopentene, 3-isopropenyl-5,5-dimethyl-	-	-	-	-	-	-	-	0.33	-	-
74	1,3-Dioxol-2-one	-	-	-	-	-	-	-	0.20	-	-
75	Allyl fluoride	-	-	-	-	-	-	-	0.06	-	-
76	Formic acid	-	-	-	-	-	-	-	11.32	-	-
77	1-Undecene	-	-	-	-	-	-	-	0.15	-	-
78	1,3-Butadiene, 2-methyl-	-	-	-	-	-	-	-	-	-	0.18
79	2-Fluoropropene	-	-	-	-	-	-	-	-	-	0.05
80	Butane, 1-[(2-methyl-2-	-	-	-	-	-	-	-	-	-	0.03

	propenyl)oxy]-										
81	Cyclohexane	-	-	-	-	-	-	-	-	-	0.74
82	Dimethyl sulfide	-	-	-	-	-	-	-	-	-	0.11
83	Methylene chloride	-	-	-	-	-	-	-	-	-	0.12
84	N-Propyl-3,4-methylenedioxyamphetamine	-	-	-	-	-	-	-	-	-	0.03
85	Pentane, 3-ethyl-2,2-dimethyl-	-	-	-	-	-	-	-	-	-	0.08
86	Tris(trimethylsilyl)borate	-	-	-	-	-	-	-	-	-	0.04
87	Ethanol, 2-methoxy-	-	-	-	-	-	-	-	-	-	0.39

Table S7 The type and contents of volatile components in bamboo, the staple food of giant panda metabolite profiles.

**Bamboo species**	**Quantity/content**	**alkanes**	**alkenes**	**aromatics**	**alcohols**	**phenol**	**aldehydes**	**ethers**	**ketones**	**acids**	**esters**	**heterocyclic**
Bashania fargesii	Number of components	5	10	1	9	1			3	1	2	1
	Relative content（%）	26.25	32.79	0.37	14.76	0.1			2.3	11.00	2.67	0.22
Indocalamus tessellatus	Number of components	3	1	2	15	1	1	1	2	2		3
	Relative content（%）	13.12	0.14	11.37	56.49	0.82	0.25	0.53	0.36	2.4		1.52
Pseudosasa	Number of components	11		1	18	1	2	1	1	1		

japonica	Relative content（%）	12.11		2.76	69.3	0.32	1.51	2.27	0.26	0.56		
Phyllostachys bissetii	Number of components	2		2	17	1		2	4	1		1
	Relative content（%）	0.95		25.83	59.52	1.32		0.82	2.72	1.75		0.16
Phyllostachys aureosulcata	Number of components	1	3	2	13	1			2			1
	Relative content（%）	0.20	2.47	14.16	75.43	0.61			3.07			0.40
Phyllostachys vivax	Number of components	1	2	2	14	1	1	1	2	1		
	Relative content（%）	0.59	0.56	20.98	56.07	1.29	0.31	0.32	2.03	1.86		
Phyllostachys propinqua	Number of components	3	3	2	16	1	2		2			2
	Relative content（%）	1.47	14.47	8.93	58.59	0.70	0.91		1.06			9.29
Phyllostachys parvifolia	Number of components		5	2	16	1	1	1		1		2
	Relative content（%）		12.47	5.75	68.96	1.29	0.14	0.47		1.25		0.33
Phyllostachys iridescen	Number of components		1	2	16	1	1	2		1		1
	Relative content（%）		0.5	13.39	64.88	0.94	0.53	1.03		0.64		0.22
Phyllostachys praecox	Number of components		9	2	17	1	1	1		1	2	2
	Relative content（%）		1.41	8.78	79.82	0.88	0.09	0.27		0.08	0.45	0.25

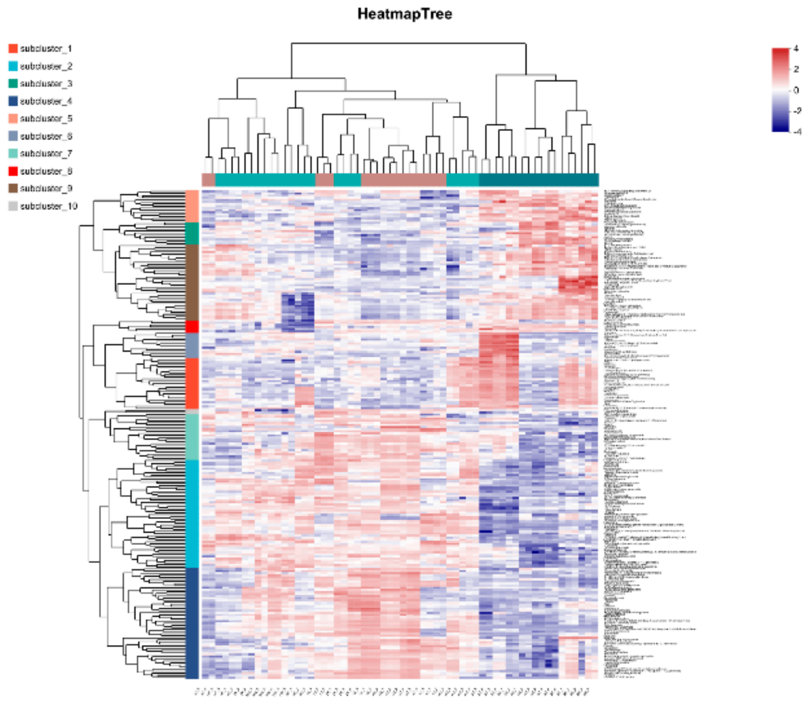

Figure S1. Heat map showing the clustering of metabolites that differ significantly in abundance between the preferred bamboo shoots and inedible shoots. Each column represents a sample, and each row represents a metabolite. The color represents the relative abundance of the metabolite in this group of samples. The tree on the left clusters the metabolites (named on the right) and the closer two branches, the closer they are in abundance. The upper tree clusters the samples (named at the bottom) and the closer two branches, the closer they are in metabolite profiles.

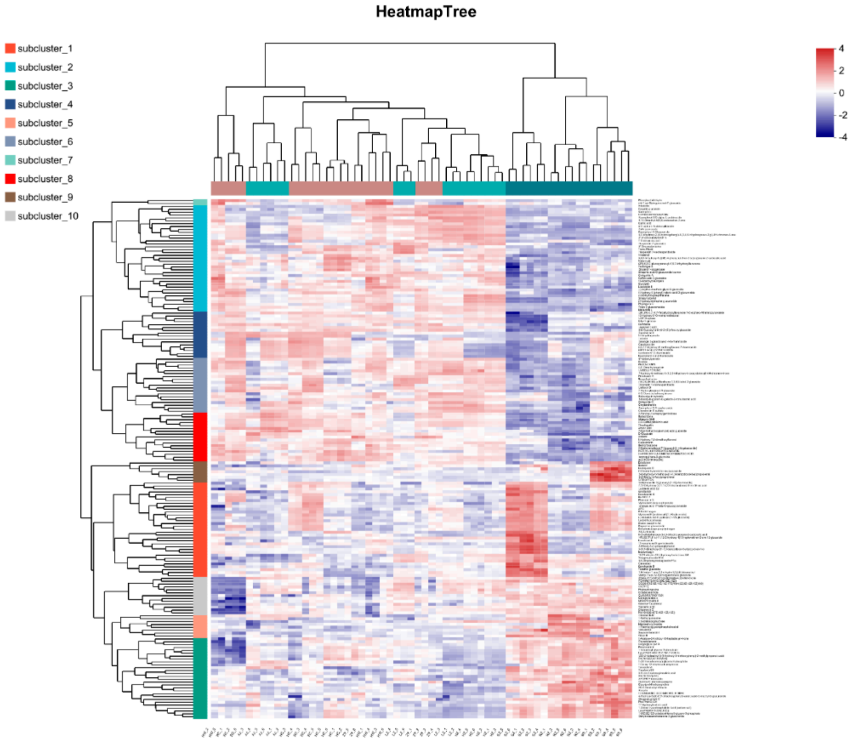

Figure S2. Heat map showing the clustering of metabolites that differ significantly in abundance between the preferred bamboo shoots and edible shoots. Each column represents a sample, and each row represents a metabolite. The color represents the relative abundance of the metabolite in this group of samples. The tree on the left clusters the metabolites (named on the right) and the closer two branches, the closer they are in abundance. The upper tree clusters the samples (named at the bottom) and the closer two branches, the closer they are in metabolite profiles.

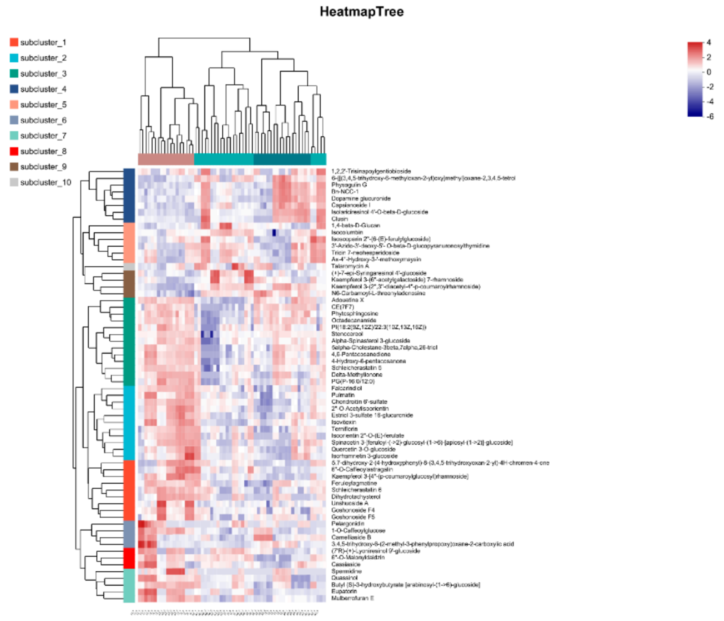

Figure S3. Heat map showing the clustering of metabolites that differ significantly in abundance between the edible and inedible bamboo shoots. Each column represents a sample, and each row represents a metabolite. The color represents the relative abundance of the metabolite in this group of samples. The tree on the left clusters the metabolites (named on the right) and the closer two branches, the closer they are in abundance. The upper tree clusters the samples (named at the bottom) and the closer two branches, the closer they are in metabolite profiles.
